# An Injectable Hydrogel Scaffold Loaded with Dual-Drug/Sustained-Release PLGA Microspheres for the Regulation of Macrophage Polarization in the Treatment of Intervertebral Disc Degeneration

**DOI:** 10.3390/ijms24010390

**Published:** 2022-12-26

**Authors:** Haozhe Cheng, Qian Guo, Hongjian Zhao, Kun Liu, Honglei Kang, Fang Gao, Jianfeng Guo, Xi Yuan, Shuang Hu, Feng Li, Qin Yang, Zhong Fang

**Affiliations:** 1 Department of Orthopedics, Tongji Hospital, Tongji Medical College, Huazhong University of Science and Technology, 1095 Jiefang Avenue, Wuhan 430030, China; 2 State Key Laboratory of Advanced Technology for Materials Synthesis and Processing, Biomedical Materials and Engineering Research Center of Hubei Province, Wuhan University of Technology, Wuhan 430070, China; 3 Department of Pathology, Tongji Hospital, Tongji Medical College, Huazhong University of Science and Technology, 1095 Jiefang Avenue, Wuhan 430030, China

**Keywords:** drug delivery, hydrogel, intervertebral disc degeneration, immune regulation, kartogenin

## Abstract

Due to the unique physical characteristics of intervertebral disc degeneration (IVDD) and the pathological microenvironment that it creates, including inflammation and oxidative stress, effective self-repair is impossible. During the process of intervertebral disc degeneration, there is an increase in the infiltration of M1 macrophages and the secretion of proinflammatory cytokines. Here, we designed a novel injectable composite hydrogel scaffold: an oligo [poly (ethylene glycol) fumarate]/sodium methacrylate (OPF/SMA) hydrogel scaffold loaded with dual-drug/sustained-release PLGA microspheres containing IL-4 (IL-4-PLGA) and kartogenin (KGN-PLGA). This scaffold exhibited good mechanical properties and low immunogenicity while also promoting the sustained release of drugs. By virtue of the PLGA microspheres loaded with IL-4 (IL-4-PLGA), the composite hydrogel scaffold induced macrophages to transition from the M1 phenotype into the M2 phenotype during the early induced phase and simultaneously exhibited a continuous anti-inflammatory effect through the PLGA microspheres loaded with kartogenin (KGN-PLGA). Furthermore, we investigated the mechanisms underlying the immunomodulatory and anti-inflammatory effects of the composite hydrogel scaffold. We found that the scaffold promoted cell proliferation and improved cell viability in vitro. While ensuring mechanical strength, this composite hydrogel scaffold regulated the local inflammatory microenvironment and continuously repaired tissue in the nucleus pulposus via the sequential release of drugs in vivo. When degenerative intervertebral discs in a rat model were injected with the scaffold, there was an increase in the proportion of M2 macrophages in the inflammatory environment and higher expression levels of type II collagen and aggrecan; this was accompanied by reduced levels of MMP13 expression, thus exhibiting long-term anti-inflammatory effects. Our research provides a new strategy for promoting intervertebral disc tissue regeneration and a range of other inflammatory diseases.

## 1. Introduction

Intervertebral disc degeneration is a common clinical degenerative disease that can seriously affect the life and work of patients while also creating a heavy economic burden for families and society. Traditional conservative and surgical treatments can only relieve the symptoms of this disease; however, the effects of such treatments are not satisfactory [1]. Over recent years, an increasing body of evidence has proven that the surgical treatment of intervertebral disc degeneration can accelerate the development of adjacent intervertebral disc degeneration. Therefore, the in situ bioremediation of intervertebral disc degeneration has become an ideal goal for clinicians and researchers [2,3].

Synthetic hydrogel has a good prospect in intervertebral disc tissue engineering and regenerative medicine, and it has several advantages, such as easy access to raw materials, low price, and good controllability of key properties [4]. Transdermal illumination and irradiation through blood vessel walls have both been used in situ to produce minimally invasive photopolymerization [5]. The modification of oligo [poly(ethylene glycol) fumarate] (OPF) polymer hydrogel scaffolds with polyethylene glycol (PEG) creates a hydrogel with a macroporous three-dimensional structure that can produce a stable polymer network through chemical crosslinking with controllable mechanical strength and low immunogenicity [6,7]. However, most of the hydrogels based on PEG exhibit poor biocompatibility and are not efficient with regards to promoting cell growth and proliferation [8]. It has been reported that the attachment and proliferation of cells in biomaterials depend strongly on their physical and chemical properties, such as charge and hardness [9,10]. In a previous study, Tan et al. reported that the adhesion of osteoblast-like cells to charged hydrogels was enhanced regardless of the polarity of the charge [11]. Furthermore, other researchers have found that the expression of the protein aggregin increased significantly in negatively charged hydrogels [12]. Sodium methacrylate (SMA) can be used as a surface modifier through carbon–carbon double bonds to introduce a negative charge into the hydrogel.

The mechanical strength and immunological effects of implanted biomass materials represent significant determinants for their application in disc herniation tissue engineering [13,14]. The process of tissue regeneration is heavily restricted by disorganized macrophage activation; this eventually results in fibrous wrapping [15]. Continuous and aggravated inflammatory reactions will also lead to long-term inefficient repair or fibrosis, thus causing a further loss of organ function [16]. Macrophages are one of the key immune cells participating in the inflammatory response and play a dominant role in the recovery of a steady state in tissues [17]. Both proinflammatory (M1) and anti-inflammatory (M2) macrophages are functional subgroups of macrophages. M2 macrophages can secrete the Th2 type of immune-related cytokines, including IL-10 and IL-13. The inhibition of inflammation can also contribute to the acceptance of biomaterials implanted in tissue engineering [18].

Hydrogel-loaded drugs cannot efficiently control drug release; their combination with drug microsphere carriers can often achieve a better therapeutic effect [19]. Drug microsphere carriers need to have the characteristics of degradability and good biocompatibility [20]. PLGA microspheres are a form of copolymer composed of lactic acid and glycolic acid. Following the ternary acid cycle, these microspheres are primarily hydrolyzed in vivo and released as water and carbon dioxide. Thus, PLGA microspheres can be used to deliver functional small molecular drugs, polypeptide proteins, and other biological macromolecular drugs and vaccines [21]. Once a drug has been encapsulated in microspheres, its release, stability, and utilization can be maintained, and side effects can be reduced, thus improving patient compliance and improving the therapeutic effect [22,23]. In contrast with the traditional emulsified solvent evaporation method, the microfluidic method can control the flow rate ratio of the oil phase to water phase and the concentration of PLGA in the oil phase [24]. This allows us to regulate the size of the microspheres prepared by this method in a controllable manner but with a uniform diameter.

Functional intervertebral disc design is a key topic in tissue engineering at present [25]. Andreas Schmocker et al. studied a photopolymerized PEGDMA hydrogel for intervertebral disc degeneration [5]. Jin Bai et al. studied a reactive oxygen species-scavenging scaffold with rapamycin for treatment of intervertebral disc degeneration [18]. In the present study, we further optimized and improved OPF by the addition of a negative charge-forming agent (sodium methacrylate) and by additional polymerization to form a new charged and controllable bioactive hydrogel polyethylene glycol fumarate/sodium methacrylate (OPF/SMA) scaffold. Two types of microspheres (IL-4-PLGA and KGN-PLGA) were embedded in OPF/SMA hydrogel scaffolds to achieve the purpose of sequential drug release. IL-4 can induce the transformation of M0/M1 macrophages to M2 macrophages, reduce the secretion of proinflammatory factors (such as TNF- α, IL-6, and IL-1 β), and increase the secretion of anti-inflammatory cytokines (such as IL-10, IL-13, and TGF- β), which can promote tissue repair [26,27]. Due to the plasticity of the polarization state, it is essential to control macrophage polarization from the M1 to the M2 phenotypes in order to facilitate intervertebral disc tissue repair [28]. Kartogenin (KGN) is a stable nonprotein molecule with a structure of 2-[(4-phenylphenyl) carbamyl] benzoic acid [29]. This can enhance the homing ability of endogenous host mesenchymal stem cells (MSCs), thus allowing cartilage to be regenerated without cell transplantation [30]. KGN can also inhibit pain and inflammation by inducing IL-10 [31,32].Through a series of in vitro experiments, the morphological and physicochemical characteristics of OPF/SMA hydrogel were thoroughly characterized. Additionally, the nucleus pulposus cell was used to evaluate the biological characteristics of OPF/SMA hydrogel, such as cytocompatibility, antioxidant activity, prodifferentiation capacity, and activity to modulate the local inflammatory environment. Finally, the OPF/SMA hydrogel loaded with PLGA microspheres was implanted into a rat model of caudal intervertebral disc degeneration to evaluate its ability to repair intervertebral disc degeneration in vivo.

## 2. Results and Discussion

### 2.1. Fabrication and Characterization of OPF/SMA Composite Hydrogel Scaffold

**Figure 1:**
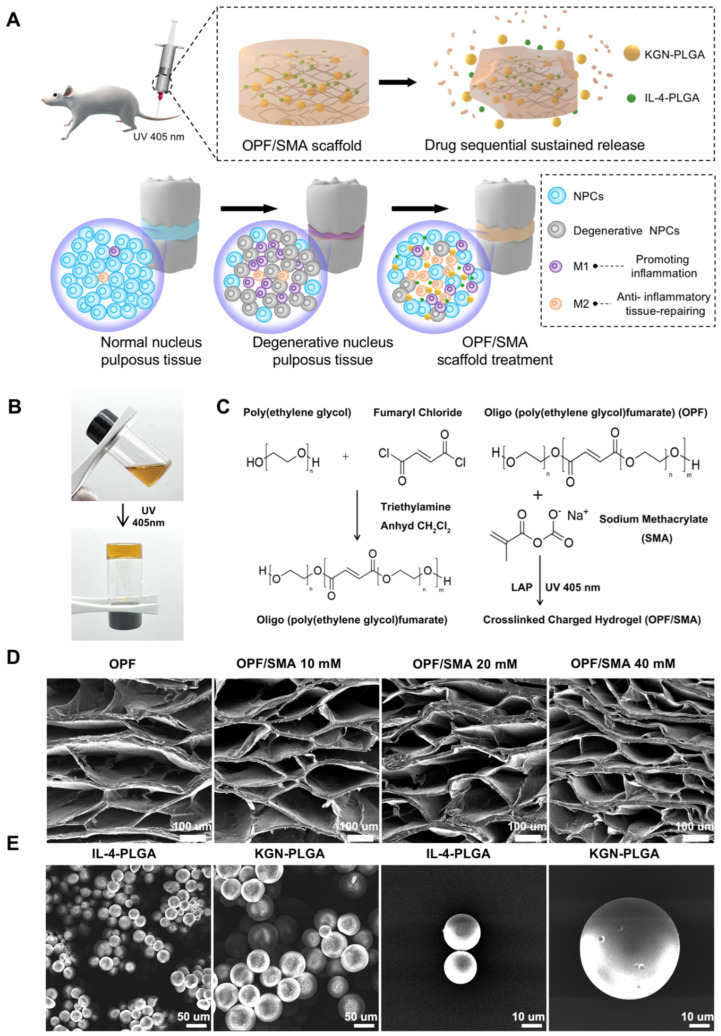
Synthesis and characterization of OPF/SMA hydrogel scaffolds and PLGA microspheres. (**A**) Schematic diagram of OPF/SMA hydrogel scaffold loaded with double-drug-loaded microspheres to regulate the microenvironment of intervertebral disc inflammation. (**B**) After the precursor solution of OPF/SMA was irradiated by ultraviolet light of 405 nm for 20 s, the carbon–carbon double bond broke and then reconnected to form a hydrogel. (**C**) Schematic diagram of chemical crosslinking of OPF/SMA hydrogel scaffolds. (**D**,**E**). Scanning electron microscope (SEM) imaging of OPF/SMA, IL-4-PLGA, and KGN-PLGA. The surface of the samples was sputter-coated with gold and palladium for 60 s and then detected at 5 kV accelerating voltage.

Over recent years, various new biomedical materials and strategies have been used for local treatment and to promote the regeneration of damaged tissues [33,34,35,36]. Here, we built an OPF/SMA hydrogel scaffold formed in situ, combined with PLGA microspheres of different specifications to achieve the purpose of timed slow release. This scaffold can provide new strategies for regulating local inflammatory microenvironments to promote the regeneration of IVD tissue (Figure 1A). We chose pyrrolidone as a crosslinking agent and LAP as a photoinitiator. To promote cell adhesion and proliferation, sodium methacrylate (SMA), a negative-charge former, was grafted onto OPF hydrogel scaffolds. After UV irradiation at a wavelength of 405 nm for 20 s, the carbon–carbon double bond was broken and then reconnected to form the OPF/SMA hydrogel scaffold after crosslinking (Figure 1B,C). The purpose of the grafted SMA was to simulate the microenvironment of the nucleus pulposus tissue. The nucleus pulposus tissue in the intervertebral disc had a higher osmotic pressure due to the high negative charge of the glycosaminoglycan side chain, which not only regulated and maintained the tissue’s hydration but also regulated and maintained the intervertebral disc height. Aggrecan eventually deteriorated and vanished from the intervertebral disc during intervertebral disc degeneration. Reduced hydration and disc height resulted from this loss of fixed charge [11,37,38].

**Figure 2:**
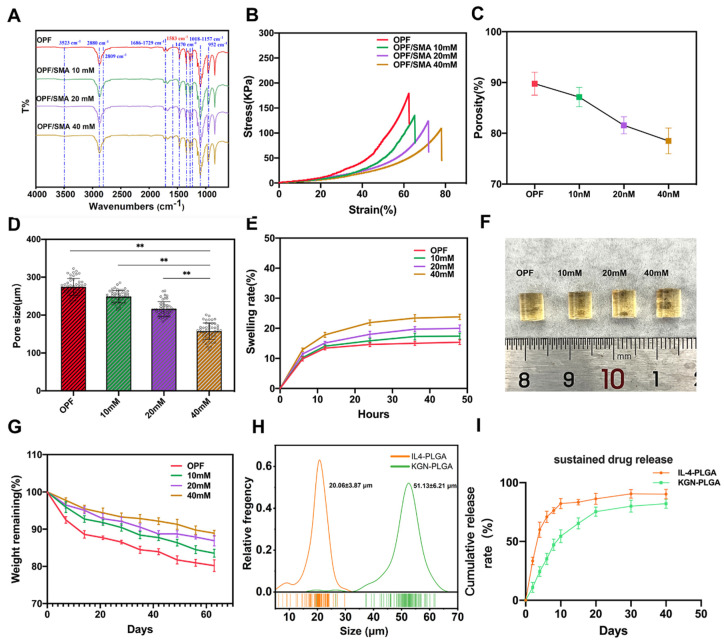
Characterization and analysis of OPF/SMA hydrogel scaffolds and PLGA microspheres. (**A**) Fourier transform infrared spectroscopy (Thermo Scientific Nicolet 6700, Waltham, MA, USA) was used to characterize the changes of functional groups on OPF and OPF/SMA. Potassium bromide was used to press the tablets in the infrared, and the infrared wavelength range was (4000–400 cm^−1^). (**B**) The compression modulus of OPF/SMA hydrogel scaffold was measured. (**C**,**D**) The porosity was calculated with the ethanol exchange method. The pore size of OPF, OPF/SMA 10 mM, OPF/SMA 20 mM, and OPF/SMA 40 mM was quantified from SEM images with ImageJ. ** *p* < 0.01. (**E**,**F**) Swelling diagram of hydrogel. Soak the hydrogel in ddH_2_O for 48 h. Statistical significance was calculated by *t*-test. (**G**) In vitro degradation curves of OPF, OPF/SMA 10 mM, 20 mM, and 40 mM hydrogels in PBS at 37 °C. (**H**) The particle size of the freeze-dried PLGA microspheres was analyzed with ImageJ software 2.3.0 (NIH, Bethesda, MD, USA) using SEM pictures. (**I**) Entrapment rate and in vitro sustained release of PLGA microspheres (*n* = 3).

In the Fourier transform infrared spectrum of OPF/SMA (Figure 2A), 1686–1729 cm^−1^ is a typical carbonyl stretching vibration peak of chain-like saturated ketones; this was the characteristic absorption peak of an ester bond, indicating that the OPF hydrogel scaffold had been synthesized successfully. The absorption peak near 1018–1157 cm^−1^ is the compound peak of the stretching vibration peak of the C–O–C ether bond with different degrees of polymerization in the molecular structure. The measurement 1583 cm^−1^ is the characteristic peak of ester carbonyl in SMA, and the intensity change of this peak is positively correlated with the concentration of the monomer.

The freeze-dried OPF/SMA hydrogel’s SEM images revealed a highly porous, linked network with micropores dispersed randomly throughout the polymer matrix (Figure 1D). Pore diameter and porosity are significant design factors for scaffolds. (Figure 1D). Pore diameter and porosity are important indices in scaffold design. While maintaining the mechanical qualities of the scaffold, an appropriate pore size and porosity can create a positive microenvironment for cell migration and proliferation [39]. The higher the proportion of negative charge-forming agent SMA grafted onto the OPF hydrogel, the smaller the porosity, which decreased from 89.2% to 77.6% (Figure 2C). The mean pore diameter of OPF was 274.6 ± 22.0 μm, and the pore diameters of the OPF hydrogel scaffold grafted with 10 mM SMA, 20 mM SMA, and 40 mM SMA were 248.9 ± 16.2 μm, 216.3 ± 19.2 μm, and 157.5 ± 21.8 μm (Figure 2D), respectively. According to prior research, scaffolds with a diameter of 100 to 200 μm are helpful for cartilage and nucleus pulposus regeneration [40,41]. Therefore, the pore size of our OPF/SMA hydrogel scaffold is suitable for intervertebral disc repair.

The results of multiple swelling tests show that all hydrogel samples had reached a swelling equilibrium after 48 h, and in the concentration range of 0–40 mM, the higher the proportion of SMA grafting, the higher the swelling ratio of OPF hydrogel. After 48 h, the hydrogel scaffold of each group reached a swelling equilibrium. The mean swelling ratio of OPF was 15.37% ± 0.76%, and the swelling ratios of OPF hydrogel scaffold grafted with 10 mM SMA, 20 mM SMA, and 40 mM SMA were 17.40% ± 1.01%, 20.00% ± 1.13%, and 23.80% ± 0.89% (Figure 2E,F), respectively. The degradation of the hydrogel scaffold in the PBS solution was then studied. The hydrogel was continuously degraded and the OPF/SMA 40 mM hydrogel with the lowest degradation rate maintained at 88.9% after 60 days, *p* < 0.05 (Figure 2G). Specifically, the early assistance of tissue formation is ensured by the stability of the hydrogel scaffold, and the delayed disintegration of the materials may make room for the development of new tissues [42].

As the intervertebral disc needs to be subjected to continuous compressive stress, the anticompression performance of an intervertebral disc repair hydrogel is particularly critical, and it is suggested that successful materials must have a high adhesion strength (~200 kPa) [43]. Interestingly, the compression test showed that with an increase in the proportion of grafted SMA, although the maximum compressive strength decreased from 179 kPa to 109 kPa, the maximum compressive fracture strain increased from 62% to 78% (Figure 2B). The reason for this result may be that the crosslinking degree increases with the increase in the proportion of SMA grafting, so that the toughness of the hydrogel is stronger. The mechanical properties of our OPF/SMA hydrogel is therefore suitable for intervertebral disc repair.

PLGA microspheres were prepared by the microfluidic method [16,27]. PLGA microspheres play an important role in accurately controlling drug release PLGA can significantly prolong the duration of drug treatment and reduce side effects. Microspheres must adhere to rigorous uniformity and particle size standards during the process of the drug delivery. The capacity to control the unique position and rate of drug release is significantly impacted by the size uniformity of the microspheres. Furthermore, the size of the microspheres needs to be adjusted to meet the drug delivery requirements of different diseases [44]. Traditional microsphere preparation methods usually have a wide particle size distribution, such as emulsification and spray drying [24]. In our study, we used the microfluidic method to prepare the PLGA microspheres. The entrapment efficiency (EE%) of drugs is shown in Table 1. The entrapment efficiency of PLGA microspheres prepared by the microfluidic method was more than 90%. The entrapment efficiency of the IL-4-PLGA microspheres was 91.94%, *p* < 0.01. The entrapment efficiency of the KGN-PLGA microspheres was 93.57%, *p* < 0.01. The freeze-dried PLGA microspheres loaded with IL-4 and KGN exhibited a relatively uniform diameter by SEM electron microscopy (Figure 1E). The particle size of the microspheres was analyzed by ImageJ (Figure 2H). The diameters of the PLGA microspheres loaded with IL-4 and the PLGA microspheres loaded with KGN were 20.06 ± 3.87 μm and 51.13 ± 6.21 μm, respectively. For the PLGA microspheres loaded with IL-4 and the PLGA microspheres loaded with KGN, the release efficiency from 0 to 40 days was measured by ELISA kits and UV spectrophotometry, respectively. Although there was an inevitable problem with regards to explosive drug release in the PLGA microspheres, and the results show that 82% of the drugs were released from the IL-4-PLGA microspheres and 54% from the KGN-PLGA microspheres in 10 days. The KGN-PLGA microspheres took 30 days to release 80% of their drug content (Figure 2I).

**Figure 3:**
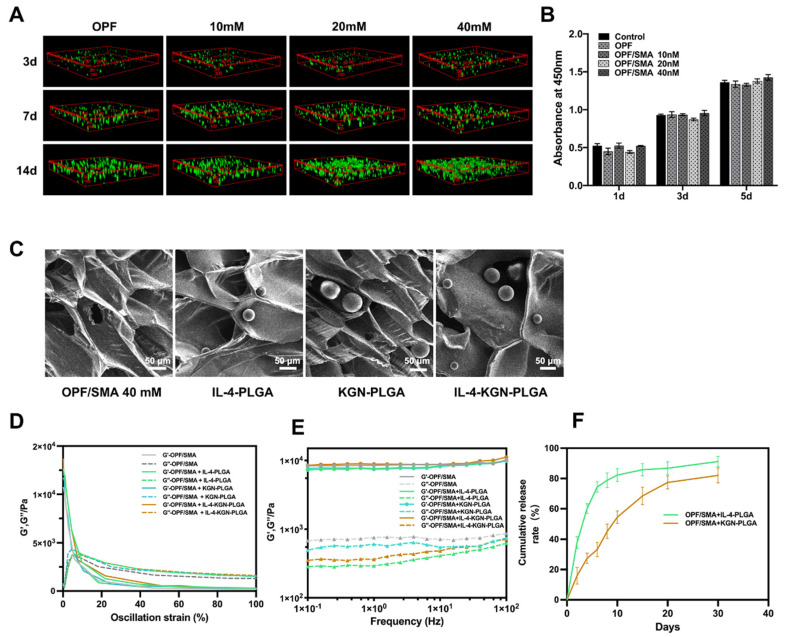
Cell proliferation and cell viability staining experiment. (**A**) 3D cell culture. The cell viability was detected by Calcein-AM/PI double staining kit. Fluorescence photos were taken by laser confocal microscope. (**B**) Cytotoxicity test and cck8 kit detection. After incubation for 1 day, 3 days, and 5 days, the cell proliferation was detected by CCK8 kit. (**C**) Scanning electron microscope (SEM) imaging of hydrogel loaded with IL-4-PLGA microspheres and KGN-PLGA microspheres. (**D**) Dynamic modulus of OPF/SMA, OPF/SMA+IL-4-PLGA, OPF/SMA+KGN-PLGA, and OPF/SMA+IL-4-KGN-PLGA at varying stains from 0.1% to 100%. (**E**) Dynamic modulus of OPF/SMA, OPF/SMA+IL-4-PLGA, OPF/SMA+KGN-PLGA, and OPF/SMA+IL-4-KGN-PLGA at varying angular frequencies from 0.1 to 100 Hz. (**F**) In vitro sustained release of OPF/SMA+IL-4-PLGA and OPF/SMA+KGN-PLGA.

The IL-4-PLGA and KGN-PLGA were loaded into the hydrogel. Scanning electron microscope images showed that the structure of the hydrogels did not change significantly after embedding IL-4-PLGA, KGN-PLGA, and IL-4-KGN-PLGA microspheres (Figure 3C).Then, we conducted the viscoelastic properties to evaluate the flow and deformation of the prepared hydrogel loaded with IL-4-PLGA, KGN-PLGA, and IL-4-KGN-PLGA under external force. From the strain sweeps, the incorporation of the PLGA microsphere did not change the linear viscoelastic region (Figure 3E). The storage modulus (G′) at 0.1–100 Hz of OPF/SMA 40mM, OPF/SMA+IL-4-PLGA, OPF/SMA+KGN-PLGA, and OPF/SMA+IL-4-KGN-PLGA was higher than the loss modulus (G′′), indicating the stable mechanical strength of the hydrogels (Figure 2F). The PLGA microspheres were physically mixed into the hydrogels, but few previous studies have done further studies on the viscoelasticity of hydrogels embedded with PLGA microspheres. In addition, from the results of our rheological experiments, the addition of PLGA did not significantly change the mechanical properties of the hydrogel. These findings indicate that we had achieved our expected goal in terms of the sequential sustained release of drugs.

### 2.2. The Cytocompatibility of the OPF/SMA Composite Hydrogel Scaffold

We first evaluated the cytotoxicity of OPF/SMA hydrogel scaffolds over time by way of the CCK8 test. The analysis revealed that there were no appreciable variations in nucleus pulpous cell proliferation. On the first day, the third day, and the fifth day in the complete medium and in OPF and OPF/SMA (10 mm, 20 mm, and 40 mm medium), the results show that the degradation products of the OPF/SMA hydrogel scaffold were not toxic to nucleus pulpous cells in vitro and did not significantly affect cell proliferation (Figure 3B).

Next, we cultured nucleus pulpous cells in the three-dimensional OPF/SMA hydrogel scaffold and stained the nucleus pulpous cells to evaluate the effect of the hydrogel scaffold on cell proliferation (Figure 3A). After 3 days, 7 days, and 14 days, live/dead staining assays were performed. Fluorescence photographs were taken by laser confocal microscopy, and the proliferation of cells in the hydrogel scaffold was analyzed. The mean fluorescence intensity of the nucleus pulpous cells was measured by ImageJ. On the 14th day, the proliferation of cells in the OPF/SMA 20 mM group and the 40 mM group was better; the addition of the negative charge-forming agent sodium methacrylate can better simulate the intervertebral disc microenvironment and promote cell proliferation in hydrogel scaffolds [11,12,37].

Our experimental results show that the OPF hydrogel grafted with SMA exhibited better cytocompatibility, which not only produced OPF with good mechanical properties, but also improved the disadvantages of poor cytocompatibility. The proliferation of nucleus pulposus cells in the OPF/SMA 40 mM hydrogel scaffold was the best and had better cytocompatibility. Subsequent cocultures and animal experiments were carried out with the OPF/SMA 40 mm hydrogel scaffold.

### 2.3. Regulation of Intervertebral Disc Immunity by the OPF/SMA Hydrogel Scaffold Loaded with IL-4-PLGA Microspheres

It has been reported that intervertebral disc regeneration involves angiogenesis, macrophage infiltration, and inflammation [45]. During the process of intervertebral disc degeneration, the number of resident macrophages in the nucleus pulposus increases; this is accompanied by an increase in the proportion of proinflammatory M1 macrophages and a decrease in the proportion of anti-inflammatory M2 macrophages. IL-4 can induce M0 and M1 macrophages to polarize to the M2 form, decrease the release of proinflammatory cytokines, such as TNF- and iNOS, and increase the release of anti-inflammatory cytokines, such as IL-10 and IL-13, and delay the progression of inflammation [26,45]. Therefore, it is critical to design a material that can not only regulate the local inflammatory environment but also promote regeneration, especially when the spontaneous regeneration of the intervertebral disc nucleus pulposus is insufficient [27,46,47]. The regeneration of nucleus pulposus cell is hampered, and the degradation extracellular matrix is accelerated by the excessive expression and release of inflammatory factors in the local microenvironment following injury, such as IL-1β.

**Figure 4:**
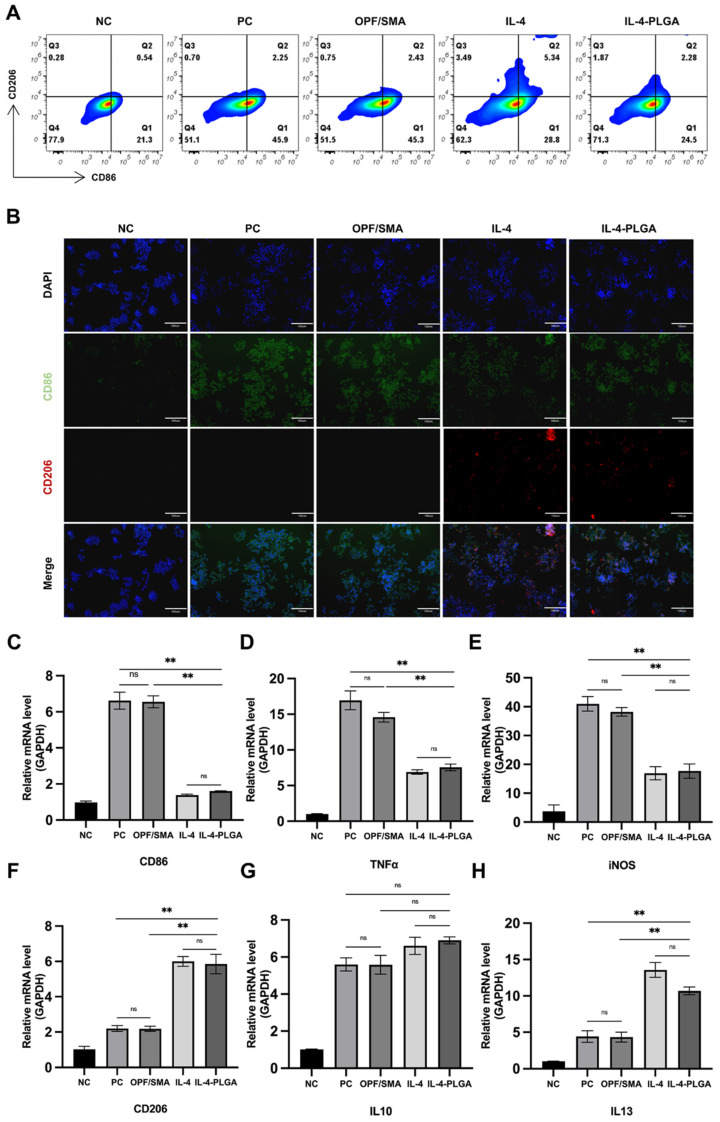
Polarization of macrophages. Raw264.7 macrophages in the control group were treated with lipopolysaccharide (100 ngmL^−1^) for 6 h and then cultured in complete culture medium. The experimental group was treated with lipopolysaccharide for 6 h and then treated with OPF/SMA-, OPF/SMA+IL4-, and OPF/SMA+IL-4-PLGA-conditioned medium. (**A**) After 24 h, the expression of CD206 and CD86 was detected by flow cytometry. (**B**) Immunofluorescence assay to detect the expression of CD206 and CD86. (**C**–**H**). Quantitative real-time reverse transcriptase-polymerase chain reaction (qRT-PCR). Statistical significance was calculated by one-way ANOVA. ** *p* < 0.01, ns > 0.05.

Based on the above, we designed poly (lactic-co-glycolic acid) (PLGA) microspheres loaded with IL-4 to reduce the immunogenicity of hydrogels and to improve the immune microenvironment of intervertebral discs. A flow cytometry analysis was performed 24 h later (Figure 4A). As expected, the macrophages treated with LPS showed a typical M1 phenotype. There was no significant change in the phenotype of the macrophages in the simple hydrogel group. The expression of CD206 in the macrophages increased, and the expression of CD86 decreased in the OPF/SMA+IL-4 and OPF/SMA+IL-4-PLGA groups, thus indicating that the PLGA microspheres loaded with IL-4 could inhibit LPS-induced inflammation and M1 polarization while enhancing the polarization of M2 macrophages. We also performed cellular immunofluorescence, which proved that IL-4-PLGA microspheres could regulate the polarization of macrophages to the M2 form (Figure 4B). We further detected the changes of inflammatory cytokines in the control group and the intervention group (Figure 4C–H). The results show that the OPF/SMA+IL-4-PLGA group increased the expression of anti-inflammatory cytokines (TNF-α, iNOS) and decreased the release of proinflammatory cytokines (IL-13). However, compared with the control group, the expression of IL-10 increased in the intervention group although there was no statistical difference. This may indicate that IL-10 is expressed in both M1 and M2 macrophages.

The experimental results show that after IL-4 was loaded on PLGA microspheres, the physiological effect of IL-4 was not significantly affected. IL-4-PLGA can induce macrophages to differentiate into the M2 type and increase the release of anti-inflammatory cytokines. This initially achieves our hypothesis of reducing the immunogenicity of the material and improving the immune microenvironment of the intervertebral disc.

### 2.4. The Effect of Sequential Drug Release from the OPF/SMA Hydrogel Scaffold on the Repair of Degenerative Nucleus Pulposus Cells

**Figure 5:**
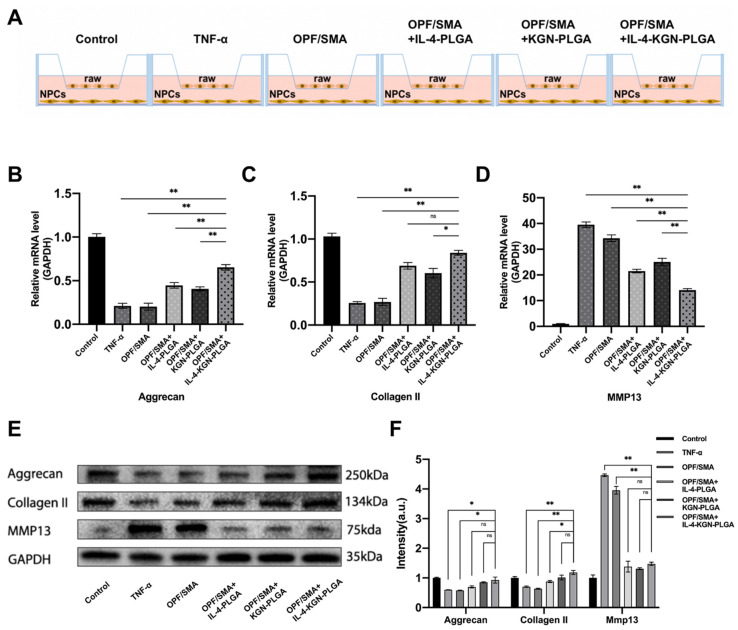
(**A**) The upper chamber of transwell is 1 × 10^5^ raw264.7, and the lower layer is 1 × 10^5^ nucleus pulposus cells. After the intervention of TNF-α 50 ng/mL for 6 h, the conditional medium replaced with hydrogel was intervened for 24 h. (**B**–**D**) Quantitative real-time reverse transcriptase-polymerase chain reaction (qRT-PCR). (**E**,**F**) Western blot detection. Grayscale analysis of protein bands. Statistical significance was calculated by two-way ANOVA. * *p* < 0.05, ** *p* < 0.01, ns > 0.05.

To study the effect of sequential drug release from the OPF/SMA hydrogel scaffold on the repair of degenerative nucleus pulposus cells, OPF+SMA, OPF/SMA+IL-4-PLGA, OPF/SMA+KGN-PLGA, and OPF/SMA+IL-4-KGN-PLGA hydrogel scaffold bead-conditioned medium successfully repaired degenerative nucleus pulposus cells in a coculture system (Figure 5A). TNF-α can be used to induce intervertebral disc degeneration [48]. The degeneration of nucleus pulposus cells was induced by TNF-α 50 ng/mL for 6 h. The extracellular matrix of degenerative nucleus pulposus cells showed a reduction in proteoglycans and type II collagen and an increase in matrix metalloproteinase MMP13 [49]. The RNA expression of aggrecan, collagen II, and MMP13 in nucleus pulposus cells was detected by q-PCR (Figure 5B–D). The results show that the expression of extracellular matrix Aggcrecan and collagen II in the nucleus pulposus was significantly up-regulated in OPF/SMA+IL-4-KGN-PLGA. The RNA expression of the extracellular matrix in the IL-4-PLGA group and KGN-PLGA group was also increased to some extent when compared with the TNF-α group. In vitro cell experiments revealed that the OPF/SMA hydrogel scaffold group did not show any anti-inflammatory repair at the RNA level. The protein expression of aggrecan, type II collagen, and MMP13 in nucleus pulposus cells was detected by Western blotting (Figure 5E,F). The expression of extracellular matrix Aggcrecan and type II collagen in the nucleus pulposus was significantly upregulated in OPF/SMA+IL-4-KGN-PLGA. The protein expression of the extracellular matrix in the IL-4-PLGA group and KGN-PLGA group was also increased when compared with the TNF-α group. The OPF/SMA hydrogel scaffold group did not show any anti-inflammatory repair in the in vitro cell experiments. Taken together, these data indicate that both q-PCR and Western blotting analysis demonstrated the exceptional ability of OPF/SMA+IL-4-KGN-PLGA hydrogel scaffoldto repair degenerative nucleus pulposus cells in the inflammatory environment.

### 2.5. The Reparative Effect of the OPF/SMA Hydrogel Scaffold on Degenerative Intervertebral Discs in Rats In Vivo

**Figure 6:**
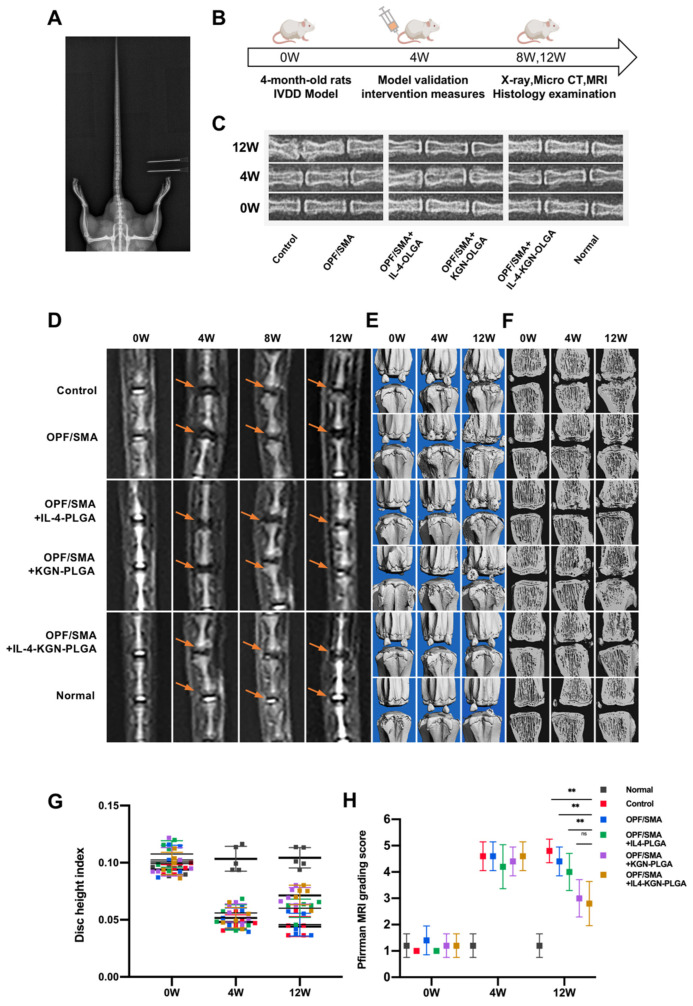
Therapeutic effect of OPF/SMA composite hydrogel in vivo. (**A**,**B**) Animal model and intervention time point. (**C**) Rat intervertebral discs can be seen on X-rays. Eight weeks following modeling. (**D**–**F**) At 4 and 8 weeks after modeling, MRI analysis and Micro-CT examination of the rat intervertebral disc were performed. (**G**,**H**) Eight weeks following surgery, changes in the caudal intervertebral DHI and Pfirrman MRI grading score of the caudal intervertebral disc in rats. The mean ± SD (*n* = 5) was used to express data. Two-way ANOVA was used to calculate statistical significance. ** *p* < 0.01, ns > 0.05.

We used an SD rat model of IVD degeneration induced by acupuncture to test the suggested strategy’s ability to promote IVD regeneration, and we assessed its effectiveness in vivo. (Figure 6A,B). The SD rats were randomly divided into three groups. Two intervertebral discs were intervened in each group. The C7/C8 and C8/C9 intervertebral discs were selected for intervention in each rat. We performed the following interventions: puncture, OPF/SMA, OPF/SMA+IL-4-PLGA, OPF/SMA+KGN-PLGA, and OPF/SMA+IL-4-KGN-PLG, nonintervention. To assess the regeneration of IVD after various therapies, X-ray exams, magnetic resonance imaging (MRI), and microcomputer tomography (Micro-CT) were used (Figure 6C–F). After 4 weeks, the MRI results demonstrated IVD degeneration in the rats’ tails. (Figure 3D). After 12 weeks, the intervertebral disc index (DHI%) was as follows: Normal (10.43%), Control (4.41%), OPF/SMA (4.58%), OPF/SMA+IL-4-PLGA (6.01%), OPF/SMA+KGN-PLGA (6.01%), and OPF/SMA+IL-4-KGN-PLGA (7.10%). The results of the X-ray show that the intervertebral disc height index in the OPF/SMA+IL-4-KGN-PLGA treatment group was better than that in the degeneration control group, *p* < 0.01. The therapeutic effect of the simple OPF/SMA group was not obvious. The therapeutic effect of the OPF/SMA+IL4-PLGA group was also not obvious when compared with the control group, *p* > 0.05. The therapeutic effect of the OPF/SMA+KGN-PLGA group was better than that of OPF/SMA, *p* < 0.05 (Figure 6C,G). In the OPF/SMA+IL-4-KGN-PLGA group, the T2-weighted signal of the MRI was relatively enhanced at 12 weeks. Furthermore, compared to the control group, single immunomodulatory therapy, and single KGN therapy, the Pfirman MRI score was significantly lower, *p* < 0.01. The results of the Micro-CT are consistent with those of the X-ray and MRI, and it was observed that the osteophyte in the treatment group was less than that in the control group (Figure 6E,F).

**Figure 7:**
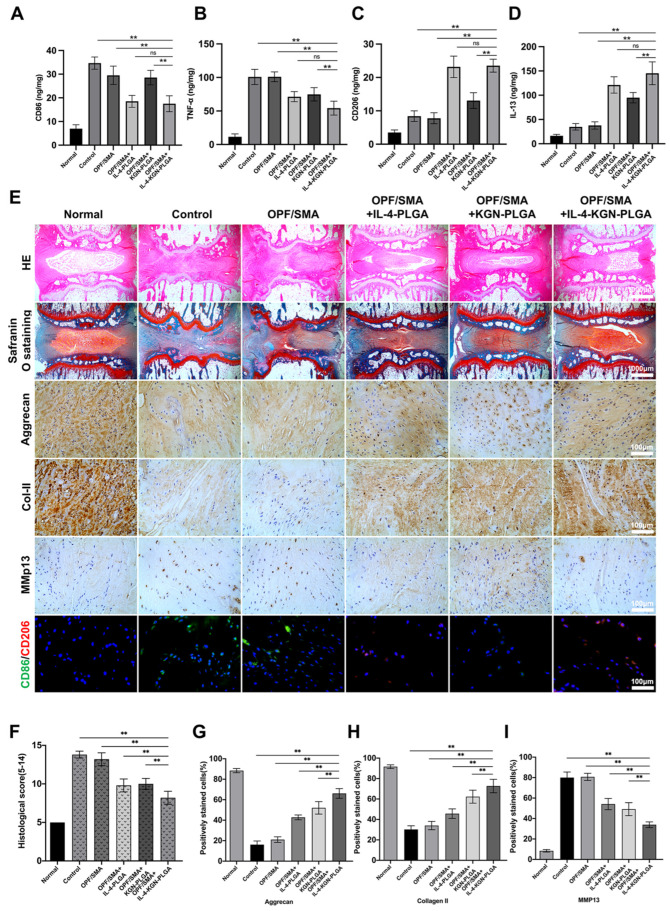
(**A**–**D**). The levels of CD86, TNF-α, CD206, and IL-13 in rat caudal intervertebral discs were detected by ELISA after two weeks of treatment (*n* = 5). (**E**) The normal group, the control group, and the experimental group were subjected to H&E staining (Scale bars, 1000 µm), safranin o-fast green staining (Scale bars, 1000 µm), immunohistochemistry (Scale bars, 500 µm), and CD206/CD86 immunofluorescence assay (Scale bars, 500 µm). The mean ± SD (*n* = 5) was used to express data. Statistical significance was calculated by *t*-test. (**F**) Histological score of rat tail intervertebral disc. The intervertebral disc histology scores in the normal group, the control group, and the experimental group at 12 weeks following surgery. (**G**–**I**) The quantitative analysis of positively stained cells for Aggrecan, Collagan II, and MMP13 in samples. The one-way ANOVA was used to calculate statistical significance. ** *p* < 0.01, ns > 0.05.

To determine whether hydrogels loaded with IL-4-PLGA can inhibit M1 macrophages or promote M2 macrophages in the early stage, the levels of CD86, TNF-α, CD206, and IL-13 in rat caudal intervertebral discs were detected by ELISA after two weeks of treatment (Figure 7A–D). Compared with the control group, the levels of CD206 and IL-13 were upregulated, and the levels of CD86 and TNF-α were downregulated in OPF/SMA+IL-4-PLGA and OPF/SMA+IL-4-KGN-PLGA, *p* < 0.01. There was no significant difference between the control group and the OPF/SMA group. In the OPF/SMA+KGN-PLGA group, the specific indexes of macrophages, CD206, and CD86 did not change significantly. This suggests that the early sustained release of IL-4 microspheres promotes the polarization of macrophages in the intervertebral disc to M2.

The intervertebral disc tissue was detected by immunohistochemistry (Figure 7E). Histochemical scoring showed that the OPF/SMA+IL-4-KGN-PLGA combined with the hydrogel scaffold group had the best therapeutic effect (Figure 7F). Compared with the control group, HE staining and safranine O-fast green staining showed that the intervertebral disc cartilage endplate was intact and that the structural destruction and tissue deterioration were greatly reduced in the OPF/SMA+IL-4-KGN-PLGA compound hydrogel scaffold group. The results show that a local injection of OPF/SMA+IL-4-KGN-PLGA combined with hydrogel scaffold reduced the degeneration of IVD in rats. However, the effect of single treatment in the OPF/SMA+IL-4-PLGA group and the OPF/SMA+KGN-PLGA group was not as good as that in the combined treatment group. In the OPF/SMA+IL-4-KGN-PLGA complex hydrogel scaffold group, the expression of Aggrecan and COL2 was upregulated, while the expression of MMP13 was downregulated, *p* < 0.01. (Figure 7G-I). The results of the immunofluorescence analysis show that compared with the denatured control group, the expression of CD206 in the experimental group was upregulated, and the expression of CD86 was downregulated, thus showing that the compound hydrogel scaffold played a role in immune regulation.

Therefore, our results show that the OPF/SMA+IL-4-KGN-PLGA hydrogel scaffold injection can protect intervertebral disc degeneration and even promote tissue regeneration in injured segments through the early regulation of the inflammatory microenvironment and the continuous anti-inflammatory repair of IVDD. The effect of single immune regulation was not obvious; this might be due to the poor immune environment of the intervertebral disc itself, the low proportion of resident macrophages, and the lack of vascular infiltration; it is difficult for blood-derived immune cells to enter the intervertebral disc [2,45,50]. However, the role of immune regulation cannot be ignored and can be used as an adjuvant treatment for intervertebral disc regeneration medicine. The effect of combined therapy will be more obvious.

Although the evidence could support our conclusion, there are still several limitations in this study. The disorder of the internal environment of intervertebral disc degeneration is a complex process, despite the fact that TNF-α can simulate the inflammatory effects of nucleus pulposus cells to some extent. In addition, the animal experiment was performed using a puncture injury animal model, which has characteristics that are different from those associated with the complex pathological process of human disc degeneration. Because chronic human intervertebral disc degeneration involves collagen disorganization and nutritional deficits, these factors are not reflected in the rat model of caudal intervertebral disc degeneration. Finally, in the process of intervertebral disc degeneration, we believe that it is meaningful to further explore the molecular mechanism and signal pathway of macrophage polarization, which will be the focus of future research.

## 3. Materials and Methods

### 3.1. Reagents and Antibodies

Polyethylene glycol (PEG) with a mean molecular weight of 4000 Da, triethylamine, fumaryl chloride, N-vinyl pyrrolidinone, blue light initiator (LAP), and sodium methacrylate were obtained from Aladdin (Shanghai, China). R&D Systems (Minneapolis, MN, USA) provided recombinant rat tumor necrosis factor (TNFα) and recombinant rat/mouse IL-4. Kartogenin was purchased from Selleck (Shanghai, China). Western blot antibodies against GAPDH (10494-1-AP, Proteintech, Wuhan, China), collagen II (ab188570, Abcam, Cambridge, UK), aggrecan (138880-1-AP, Proteintech, Wuhan, China), and MMP13 (18165-1-AP, Proteintech, Wuhan, China). CCK8 kits and a Calcein-AM/PI Double Stain Kit Calcein-AM/PI were purchased from Yeasen (Wuhan, China). DMEM/F12 medium and DMEM medium were purchased from Gibco (New York, NY, USA). The immunofluorescent antibodies included anti-CD86 (13395-1-AP, Proteintech, Wuhan, China), anti-CD206 (60143-1-Ig, Proteintech, Wuhan, China), CoraLite594 (SA00013-3, Proteintech, Wuhan, China), and CoraLite488 (SA00013-2, Proteintech, Wuhan, China). The flow cytometry antibodies included anti-CD206-PE (141705, Dakewe, Beijing, China) and anti-CD86-PE/CY7 (105013, Dakewe, Beijing, China).

### 3.2. Preparation of the OPF/SMA Hydrogel Scaffold

OPF was prepared from polyethylene glycols from an initial molecular weight of 4000 Da [51,52]. In brief, 50 g of polyethylene glycol ester underwent boiling distillation with toluene to remove residual moisture and was then dissolved in distilled dichloromethane. The polyethylene glycol obtained was then placed in an ice bath and covered with nitrogen for 10 min. Then, we added 0.9 mol of triethylamine (TEA; Aladdin, Shanghai, China) and 1.8 mol of distilled fumaryl chloride (Acros; Shanghai, China)/mol of PEG. Then, we removed the reaction container from the ice bath, stirred at room temperature for 48 h, and then removed the dichloromethane with a rotating evaporator. The OPF generated was dissolved in acetate and filtered to remove salt following reactions with TEA and chloride. OPF was then recrystallized in ethyl acetate and vacuum-dried overnight. OPF (0.2 g/mL), N-vinyl pyrrolidinone (NVP, 0.01 g/mL; Aladdin, Shanghai, China), and Lithium Phenyl (2,4,6-trimethylbenzoyl)phosphinate (LAP, 0.001 g/mL; Aladdin, Shanghai, China) were added and mixed in PBS. Then, 0 mM, 10 mM, 20 mM, and 40 mmM methyl acrocity (SMA) were added to the hybrid solutions and mixed well. The mixed solutions were then added to a tetrafluoroethylene mold and exposed to 405 nm ultraviolet light for 20 s to generate a cylindrical body with a diameter of 10 mm and a height of 5 mm. The obtained hydrogel scaffold was then soaked in ddH_2_O and freeze-dried by lyophilization after swelling equilibrium. The samples were then sent for scanning electron microscopy (SEM).

### 3.3. Preparation of the IL-4-PLGA and KGN-PLGA Microspheres

PLGA microspheres were prepared by the microfluidic method [24,53]. The velocity of the oil phase was controlled by a microfluidic injection pump (LSP01-1B). Then, 1 wt% or 2 wt% PLGA anhydrous dichloromethane solution (Aladdin, Shanghai, China) was used as the intermediate oil phase. Next, 5 µg of IL-4 was mixed into 10 mL oil phase of 1 wt% PLGA at low temperature, and then 20 μmol of KGN was mixed into 10 mL oil phase of 2 wt% PLGA. A peristaltic pump (BT100, Baoding, China) was used to control flow rate during the water phase; 1.5% PVA aqueous solution was used as the outer aqueous phase, and the PE pipes (1 mm inner diameter) were connected to form T-shaped pipes. We set the flow rates of the microinjection and peristaltic pumps at 0.2 mL/min and 4 mL/min, respectively. PLGA droplets formed in the channel, and the prepared 1.5% PVA aqueous solution was used as a solution to collect and prepare droplets. Following collection, the volatile solvent was stirred magnetically at 250 rpm. Then, IL-4-PLGA microspheres and KGN-PLGA microspheres were obtained by repeated centrifugation (5000 rpm) and washed five times in distilled water. After removing the residual PVA, IL-4-PLGA microspheres and KGN-PLGA microspheres were obtained by freeze-drying. Then, 10 mg IL-4-PLGA and 10 mg KGN-PLGA were embedded in the 1 mL precursor solution of hydrogel before photocrosslinking.

### 3.4. Scanning Electron Microscopy (SEM)

Once lyophilized, the morphology of the OPF/SMA hydrogel scaffolds and PLGA microspheres was observed by scanning electron microscopy (JSM-IT200, Hitachi, Tokyo, Japan). The samples’ surfaces were sputter-coated with gold and palladium for 60 s and then detected at an accelerating voltage of 5 kV and WD 13.9 mm for SEM imaging. Three samples were selected for electron microscope scanning at each concentration.

### 3.5. Fourier Transform Infrared (FTIR) Spectroscopy

In order to characterize the chemical reaction between OPF and SMA, Fourier transform infrared spectroscopy (Thermo Scientific Nicolet 6700, Waltham, MA, USA) was used to characterize the changes in functional groups on OPF and OPF/SMA. The infrared wavelength range was 4000–600 cm^−1^.

### 3.6. Measurement of Pore Size, Porosity, and Particle Size

Using SEM images, ImageJ software 2.3.0 (NIH, Bethesda, MD, USA) evaluated the pore size of the freeze-dried hydrogels and the particle size of the freeze-dried PLGA microspheres. We estimated porosity with the ethanol exchange approach [53]. To determine the wet weight, we weighed the dry hydrogel (W_a_), and it was then immersed in anhydrous ethanol (W_b_). The following equation was used to determine the hydrogel’s porosity: Porosity is calculated as (W_b_ − W_a_)/ρV. V represents the dried hydrogel’s volume, and ρ represents the density of ethanol.

### 3.7. Mechanical Properties of the Hydrogel Scaffold

The compression properties of the OPF/SMA hydrogels with a radius of 10 mm and a height of 5 mm were tested on a hydraulic servo universal testing machine (MTS 810, Saint Paul, MN, USA) with a compression rate of 1 mm/min. The assessment of the rheological property was performed using a rheometer (TA DHR-2, USA). Dynamic frequency sweep was performed at 1% strain from 0.1 to 100 Hz, and dynamic strain sweep was carried out from 0.1% to 100% strain at the frequency of 1 Hz.

### 3.8. Swelling Rate of the Hydrogel Scaffold

The weight variation of the design point affected how much the hydrogel swelled (0 h, 6 h, 12 h, 36 h, and 48 h). After photocrosslinking, we weighed the OPF/SMA (500 µL, *n* = 3) to obtain the starting weight (W_c_) and then wet-weighted (W_w_) the hydrogel by submerging it in PBS at 37 °C. The equation used to obtain the swelling ratio is as follows: swelling ratio = (W_w_ − W_c_)/W_c_.

### 3.9. Degradation of Hydrogel Scaffold In Vitro

After photocrosslinking, 100 μL of hydrogel (*n* = 3) was completely submerged in 5 mL of PBS and at 37 °C and 80 rpm after freeze-drying. After reaching swelling equilibrium, we obtain the weight of the gel (D_0_) and took out the hydrogel at a designed time point and placed it overnight in ddH_2_O. Finally, we dried the surface of the hydrogel to obtain the weight of the gel (D_t_). Weight remaining (%) = D_t_/D_0_.

### 3.10. Entrapment Efficiency (EE%) and In Vitro Sustained Release of PLGA Microspheres

A total of 10 mg of lyophilized IL-4-PLGA (*n* = 5) and KGN-PLGA (*n* = 5) were placed in the PBS solution (pH = 7.4, 3 mL) at 37 °C under gentle stirring. The supernatant was collected by centrifugation. The concentration of IL-4 in the supernatant was then determined with an ELISA kit, and the concentration of KGN in the supernatant was determined by ultraviolet spectrophotometry. At a specific time point (2 d, 4 d, 6 d, 8 d, 10 d, 15 d, 30 d, and 40 d), they were extracted from the supernatant. EE (%) = M_R_/M_T_. M_R_ is the actual mass of the drug in the microsphere, and M_T_ is the theoretical mass of the drug in the microsphere.

### 3.11. Cell Culture

We obtained leukemia macrophages (raw264.7) from Cyagen Biosciences (Wuhan, China). Rat nucleus pulposus cells were extracted from the caudal intervertebral disc of 4-week-old SD rats, as previously reported [54]. The cells were then grown in DMEM high glucose or DMEM/F12 (Cyagen, Wuhan, China) with 1% penicillin–streptomycin (P/S, Cyagen, Wuhan, China) and 10% fetal bovine serum. The culture media was then replaced every two days, while the cells were maintained at 37 °C and 5% carbon dioxide. Extracts were prepared from each group according to the following steps: 400 µL of hydrogel (height: 5 mm, diameter: 10 mm) was soaked in 3 mL of medium and cultured at 37 °C for 24 h. Extraction solution was filtered via a 0.22 µm filter membrane. The culture medium was then gathered, and fetal bovine serum and P/S were added as necessary. Prior to use, the obtained media were kept at 4 °C [55].

### 3.12. Cell Proliferation and Cell Viability Staining Experiments

The precursor solutions (100 μL) of OPF and OPF/SMA (10 mM, 20 mM, and 40 mM) were passed through a 0.22 μm sterilization filter and irradiated with 405 nm ultraviolet light for 20 s, soaked in 1 mL of medium, and placed on a shaker at 100 rpm for 3 days. The conditioned medium was then used for cytotoxicity testing with a 96-well plate containing 2000 cells per well. After incubation for 1 day, 3 days, and 5 days, cell proliferation was detected by a CCK8 kit. The test medium was exchanged with working fluid and incubated at 37 °C and 5% CO_2_ for 30 min. The absorbance was then detected at 450 nm using an enzyme-labeling instrument. Then, we cultured the cells in three-dimensional OPF/SMA hydrogel scaffold and performed cell viability staining to evaluate the effect of the hydrogel scaffold on cell proliferation. Specifically, PBS was used as a solvent, and the anterior solution of the hydrogel was filtered via a 0.22 μm sterilization filter. Next, 2 × 10^5^ nucleus pulposus cells were embedded in 100μL of OPF/SMA hydrogel scaffold and cultured in 3 mL of medium for 3 days, 7 days, and 14 days. The samples were washed with PBS and then incubated with the Calcein-AM/PI double stain kit (Yeasen, China) at 37 °C and 5% CO_2_ for 15 min. Using laser scanning confocal microscopy (LSCM; Olympus, Tokyo, Japan), the samples were observed after being washed with PBS.

### 3.13. Macrophage Polarization Assay

Polarization assays were performed with raw264.7 cells in a transwell plate. The OPF/SMA hydrogel scaffold embedded with IL-4-PLGA was placed in the upper layer, and raw264.7 cells were inoculated into the lower layer with 1 × 10^5^ cells in each well. Cells were then cultured in media containing 10% fetal bovine serum, LPS (100 ng/mL), and IL-4 (100 ng/mL), and OPF/SMA and OPF/SMA loaded with IL-4-PLGA microspheres were added within a specified time period. After 48 h of culture, M1 and M2 macrophages were identified by immunofluorescence staining, flow cytometry, and qPCR.

### 3.14. Realtime Fluorescence Quantitative PCR (qRT-PCR)

A total of 48 h were spent cultivating raw264.7 and nucleus pulposus cells in the test media. Following the directions provided by the manufacturer, a reverse transcriptase kit (Yeasen, China) was used to extract total RNA. After determining the RNA concentration, reverse transcriptase was used to produce cDNA. After that, qRT-PCR was carried out using a SYBR Green qPCR Kit (Yeasen, China). The primer sequences of target genes (Qingke, Wuhan, China) are shown in Table 2. The primer sequence of aggrecan aimed at the G1-B’ and IGD domains of the aggrecan gene.

### 3.15. Western Blot (WB)

Nucleus pulposus cells and raw264.7 were cocultured with hydrogel scaffold for 48 h, and the protein expression level was detected by WB. Total protein extraction kit (Yeasen, Shanghai, China) was used to extract cell protein. After the concentration was determined by BCA protein analysis kit (Yeasen, Shanghai, China), the protein was separated by SDS-PAGE electrophoresis and transferred to PVDF. The PVDF membrane was blocked with 5% bovine serum albumin for 1 h, and the first antibody was incubated at 4 °C. The antibodies included anti-Collagen II (Proteintech, Wuhan, China), anti-Aggrecan (Abcam, Cambridge, UK), anti-MMP13 (Proteintech, China), and GAPDH (Proteintech, Wuhan, China). The blots were produced with an HRP-conjugated secondary antibody and imaged with a ChemiDoc MP Imaging System (Bio-rad, Hercules, CA, USA). ImageJ 2.3.0 was used to measure the protein band strength and normalize it to the equivalent GAPDH bands (NIH, Bethesda, MD, USA).

### 3.16. Animal Model

We obtained Sprague–Dawley rats (3 months old and weighing 450 g) from Beiente (Wuhan, China). The intake of isoflurane anesthetized 35 male rats. The rat caudal discs were located using an X-ray and digital palpation (co7/8; co8/9). To corroborate this, we numbered the vertebrae in the sacral area of the radiography. Then, the rats were randomized into two groups: a normal group (*n* = 5; no needle puncture; no injection) and a degenerative group (*n* = 30; needle puncture; no injection). In order to cause puncture injury, a 21 G needle was introduced into the tail skin through the annulus fibrosus and spun continuously for 1 min [56,57]. MRI examination was performed 4 weeks later. After making it clear that the model is successful. Two intervertebral discs were intervened in each group. The C7/C8 and C8/C9 intervertebral discs were selected for intervention in each rat. We performed the following interventions: Puncture, OPF/SMA, OPF/SMA+IL-4-PLGA, OPF/SMA+KGN-PLGA, and OPF/SMA+IL-4-KGN-PLG, non-intervention. Five microliters of material was injected through a 10 microliter microinjector (diameter 0.5 mm) into the NP tissue. Then, a 405 nm ultraviolet fiber needle (diameter 0.5 mm) irradiated the injection site for 20 s for photocrosslinking.

### 3.17. MRI and Micro-CT Evaluation

The structural changes in a sagittal T2-weighted signal were evaluated by 3.0 T clinical magnetic resonance imager (Wuhan Tongji Hospital Lianying Modern Medical Diagnosis Center). Spin echo repetition time, 2275 ms; echo time, 80 ms; number of excitations, 8; field of view, 5 cm; no phase wrap; and slice thickness, 1 mm. The Pfirrman MRI grading score was assessed by two blinded researchers as described previously [57]. Changes of caudal intervertebral disc height in rats were detected by microcomputed tomography (μ-CT, Scanco Medical, Brüttisellen, Switzerland). Voltage = 100 kV; current 0 = 98 μA; and voxel size = 10 μm. From the evaluation system integrated within the μ-CT, we were able to acquire X-rays and three-dimensional (3D) images. After treatment, the changes of rat caudal intervertebral discs were analyzed by experienced blinded researchers at 0 weeks, 4 weeks, and 12 weeks. The caudal intervertebral disc samples were then obtained after the rats were sacrificed. The intervertebral disc height index (DHI) was calculated using ImageJ software (NIH, Bethesda, MD, USA) analysis of all X-ray images.

### 3.18. ELISA Assay

Following a standardized protocol, the rat intervertebral disc tissue was ground with homogenate medium; then, the grinding solution was centrifuged at 4000 r for 10 min, and the supernatant was prepared into 10% tissue homogenate. The supernatants were stored at −80 °C for the following detection: CD86, TNF-α, CD206, and IL-13 in the tissue homogenate were analyzed by ELISA kit following the instructions of the manufacturer. CD 86 (Solarbio, Beijing, China), CD206 (Meimian, Yancheng, China), TNF-α (Abclonal, Wuhan, China), and IL-13 (Abcam, Cambridge, UK).

### 3.19. Macroscopic and Histological Assessments

The extracted intervertebral disc samples were fixed with 4% *v*/*v* paraformaldehyde for 24 h. After decalcification with EDTA decalcification solution (Servicebio, Beijing, China) for 1 month, the samples were embedded in paraffin and then sliced. The slides were stained with H&E and safranin o-fast green staining. In order to generate the histological score of IVDD, the cellular structure and histology of nucleus pulposus, annulus fibrosus, and endplate were then examined under microscope by two experienced blind histological researchers [31].There were 5 points in normal group, 6–11 points in moderate degeneration group, and 12–14 points in severe degeneration group [58].

### 3.20. Tissue Immunohistochemistry

The tissue sections of intervertebral disc were put into heated sodium citrate buffer solution (pH = 6.0) for antigen repair. The tissue sections were placed in a wet box at room temperature and sealed with 3% bovine serum albumin for 20 min. Rabbit anti-Aggrecan (Proteintech,12880-1-AP), rabbit anti-MMP13 antibody (Proteintech,18165-1-AP), and rabbit anti-Collagen II antibody (Abcam, ab6586) were incubated overnight at 4 °C. The corresponding HRP-labeled secondary antibody was added to the tissue section and was incubated at room temperature for 60 min. Hematoxylin was stained quickly, washed with pure water for 10 min, and dehydrated with ethanol gradient. We sealed the sections with resin and recorded it through scanning microscope.

### 3.21. Tissue Immunofluorescence

The paraffin sections of intervertebral disc were incubated in EDTA antigen repair buffer. An autologous fluorescence quenching agent was added to eliminate autofluorescence, and then the sections were incubated with 3% bovine serum albumin for 20 min. Rabbit anti-CD86 antibody (Proteintech, 13395-1-AP) and rabbit anti-CD206 antibody (Proteintech, 60143-1-Lg) were incubated overnight at 4 °C. Then, the second antibody was added, and the sections were incubated at room temperature without light for 60 min. The nucleus was stained rapidly with 4-amino-6-diamino-2-phenylindole (DAPI). The tissue sections were sealed with antifluorescence quenching agent and observed by fluorescence microscope.

### 3.22. Statistical Analysis

All statistical analyses were analyzed with Prism version 9.0.1 (GraphPad Software, San Diego, CA, USA). One-way ANOVA, two-way ANOVA, and t-test were used for statistical analysis. All data are expressed as mean ± standard deviation (SD). When *p* < 0.05, the difference was considered to be statistically significant.

## 4. Conclusions

In this study, we prepared a new type of OPF/SMA hydrogel scaffold with an adjustable negative charge, which has good and controllable mechanical properties and biocompatibility. As a negative charge-forming agent, sodium methacrylate endows the OPF hydrogel scaffold with a controllable ionic environment, thus enhancing the compressive strain strength of the hydrogel scaffold. This enables the hydrogel scaffold to simulate the microenvironment of intervertebral discs and improves the poor biocompatibility of polymer compounds. On this basis, we prepared two types of uniform PLGA microspheres and embedded them into the hydrogel scaffold to achieve the sequential sustained release of drugs combined with early immune regulation and continuous anti-inflammatory repair. Regulating the polarization of macrophages can improve the immune microenvironment in the intervertebral disc and further reduce the immunogenicity of the material. The experimental results show that in the treatment of intervertebral disc regeneration, single immune regulation as the main treatment may not be sufficient and that the combination of immune and anti-inflammatory drugs will have a better effect. Thus, the OPF/SMA hydrogel scaffold loaded with double microspheres prepared in this study may be used as a scaffold material to treat clinical intervertebral disc degeneration.

## Figures and Tables

**Table 1:** The entrapment efficiency (EE%) and particle size of PLGA.

	Entrapment Efficiency (EE%)	Standard Error	Particle Size (μm)
IL-4-PLGA	91.94	1.81	20.06 ± 3.87
KGN-PLGA	93.57	2.37	51.13 ± 6.21

**Table 2:** Primer sequence of target genes.

Target Gene	Species	Forward	Reverse
*GAPDH*	Mouse/Rat	CCGCATCTTCTTGTGCAGTG	CGATACGGCCAAATCGTTC
*Aggrecan*	Rat	GCTACGACGCCATCTGCTACAC	ATGTCCTCTTCACCACCCACTCC
*Collagen II*	Rat	GGAGCAGCAAGAGCAAGGAGAAG	GGAGCCCTCAGTGGACAGTAGAC
*MMP13*	Rat	ATACGAGCATCCATCCCGAGACC	AACCGCAGCACTGAGCCTTTTC
*CD86*	Mouse	ACGGAGTCAATGAAGATTTCCT	GATTCGGCTTCTTGTGACATAC
*CD206*	Mouse	CCTATGAAAATTGGGCTTACGG	CTGACAAATCCAGTTGTTGAGG
*TNF-α*	Rat	ATGTCTCAGCCTCTTCTCATTC	GCTTGTCACTCGAATTTTGAGA
*iNOS*	Mouse	TCTAGTGAAGCAAAGCCCAACA	TGATGGACCCCAAGCAAGAC
*IL-10*	Mouse	GGTTGCCAAGCCTTATCGGA	CACCTTGGTCTTGGAGCTTATT
*IL-13*	Mouse	CACACAAGACCAGACTCCCCT	TGGCGAAACAGTTGCTTTGT

## Data Availability

The data presented in this study are available on reasonable request from the corresponding author.
